# Local versus Global Time in Early Relativity Theory

**DOI:** 10.3390/e26070608

**Published:** 2024-07-18

**Authors:** Dennis Dieks

**Affiliations:** History and Philosophy of Science, Utrecht University, 3584 CC Utrecht, The Netherlands; d.dieks@uu.nl

**Keywords:** time and gravity, coordinate time and proper time, gravitational redshift, principle of equivalence

## Abstract

In his groundbreaking 1905 paper on special relativity, Einstein distinguished between local and global time in inertial systems, introducing his famous definition of distant simultaneity to give physical content to the notion of global time. Over the following decade, Einstein attempted to generalize this analysis of relativistic time to include accelerated frames of reference, which, according to the principle of equivalence, should also account for time in the presence of gravity. Characteristically, Einstein’s methodology during this period focused on simple, intuitively accessible physical situations, exhibiting a high degree of symmetry. However, in the final general theory of relativity, the a priori existence of such global symmetries cannot be assumed. Despite this, Einstein repeated some of his early reasoning patterns even in his 1916 review paper on general relativity and in later writings. Modern commentators have criticized these arguments as confused, invalid, and inconsistent. Here, we defend Einstein in the specific context of his use of global time and his derivations of the gravitational redshift formula. We argue that a detailed examination of Einstein’s early work clarifies his later reasoning and demonstrates its consistency and validity.

## 1. Introduction

In his 1905 paper introducing the special theory of relativity, Einstein begins his explanation of the new kinematics with an extensive discussion of the concept of time [1], §1 (see [2], pp. 253–274, for background to Einstein’s early work on relativity theory). As Einstein emphasizes, a mathematical description of motion has no physical meaning unless we clearly understand what we mean by “time” in concrete cases. An obvious way of addressing this issue is to link the concept of time to data provided by clocks.

However, if we look at one clock at position A and another identical clock at position B, both stationary in an inertial frame, these only provide us with the *local times* at A and B, respectively. But, to conduct physics, we should be able to describe the temporal evolution of processes extending from A to B. For this, we need more than local considerations: we should be able to compare quantities defined at different positions. This requires a common, *global time*. At this point, Einstein introduces his famous simultaneity criterion: clocks at A and B are synchronous if, according to their indications, the time taken by light to travel from A to B equals the time needed to travel back from B to A. With this concept of simultaneity in hand, we can align clocks in different places so that time becomes a global concept.

Two key points stand out in Einstein’s reasoning. First, his discussion focuses on simple and easily imaginable scenarios, using familiar concepts. Second, and importantly, there is an implicit appeal to symmetry: it is assumed that a stationary system (inertial system) exists with homogeneous, isotropic, and time-invariant spatial properties. The notion of global time introduced by Einstein’s procedure reflects the global symmetries of spacetime and derives its physical significance from them.

After 1905, Einstein began working on generalizing relativity theory to include accelerated systems and, through the principle of equivalence, the presence of a gravitational field [3,4]. In these papers, he used the same strategy as before: arguing from thought experiments in simple physical scenarios involving global symmetries to reach general conclusions. In 1907, the principle of equivalence makes its first appearance, as the hypothesis that the effects of gravity on any physical process can be understood as the results of describing such processes from a reference frame accelerated relative to an inertial system. Accordingly, it became crucial to generalize the 1905 kinematical considerations to accelerated frames of reference. As it turns out, local and global notions of time can again be introduced in such accelerated frames, by a procedure that is similar to the one used in the 1905 paper. However, there is an important difference. In an accelerated frame, clocks indicating local time do not agree with clocks showing global time: local and global clocks tick at different rates.

In his 1911 paper, Einstein returned to this issue and rederived his 1907 results with a much simpler argument. Again invoking the difference between local and global time, he made a new, empirically testable prediction: starlight will be deflected by heavy celestial bodies, a result for which the 1911 paper is still famous.

In these early papers, Einstein did not introduce global time as an arbitrary fourth coordinate. On the contrary, he often referred to his global time as the “real time” of a reference system, so that local timekeeping devices lagging behind clocks indicating global time could be said to truly go slow. However, modern discussions of general relativity emphasize that there are no a priori preferred frames or a priori given global spatiotemporal symmetries (e.g., [5,6,7]); general relativity is a “background-independent” theory. This absence, in general, of global symmetries implies that Einstein’s physically significant global time cannot be guaranteed to exist. From a modern perspective, it is sufficient for a global description to have consistent global *coordinates*. These coordinates do not require a direct physical interpretation in terms of rods and clocks.

In his *Autobiographical Notes* [8] (pp. 66–67), Einstein tells us that he struggled with the idea of coordinates that do not correspond to directly measurable spatial and temporal intervals. Indeed, even in his 1916 review paper on general relativity and in later writings, he treated what we would now consider a mere time coordinate as a physically significant global time, particularly when investigating the gravitational redshift (§22 in [9], p. 97 in [10]). From a modern point of view, Einstein’s reasoning here, and similar reasoning by his contemporaries—particularly Eddington—may well seem puzzling. Many commentators, starting with Earman and Glymour [11], have indeed claimed that Einstein’s interpretation of temporal coordinates around 1916 was confused and inconsistent and that his derivations of the redshift formula were invalid. Recent examples include [7,12], while Darrigol [5] takes a somewhat more nuanced position.

However, we will attempt to show that, not only is there a strong continuity in Einstein’s handling of “time” from 1905 to 1916 and beyond, but that the details of this continuous development clarify and justify Einstein’s 1916 (and later) reasoning.

## 2. From 1905 to 1907

In his 1905 paper ([13], p. 38), Einstein notes that the description of the motion of a material point requires the specification of the values of its coordinates as a function of time and that such a description can only have physical significance if “we are quite clear as to what we understand by “time”.

To achieve this clarity, Einstein considers clocks that are all manufactured in an identical manner and are located at different positions in an inertial system. These clocks should be constructed in such a way that they indicate the familiar time of classical mechanics. This means that a material particle moving very close to a clock, so that its position can be coordinated without problems with the clock’s indications, should obey, in first approximation, Newton’s equations of motion (in particular, the law of inertia). As pointed out in the Introduction, we thus gain access to a local time, defined at the clock’s position. In order to compare times indicated by different clocks, located at different positions, we need the notion of distant simultaneity. This notion is supplied by Einstein’s famous definition ([1], p. 894 and [13], p. 40), “Let a ray of light start at the ‘A time’ $t_{A}$ from A towards B, let it at the ‘B time’ $t_{B}$ be reflected at B in the direction of A, and arrive again at A at the ‘A time’ $t_{A}^{\prime }$. By definition, the two clocks synchronize if $t_{B}-t_{A}=t_{A}^{\prime }-t_{B}$.”

With this “Einstein synchronization” in place, a global time can be defined, relative to any inertial system. Indeed, we could place clocks throughout the inertial system in question and synchronize them all with one chosen standard clock. Statements about what time it is then acquire a global meaning. For example, “it is twelve o’clock” will now refer to the infinite set of events where all synchronized local clocks show the same local time, twelve o’clock.

The global symmetries of special relativistic inertial reference frames (due to the flatness and simple topology of special relativistic spacetime) guarantee that synchronized clocks remain in synchrony over time: external circumstances are the same everywhere, and all clocks have been constructed so as to run at the same rate under equal conditions. Therefore, a set of stationary “local clocks” in an inertial system, once synchronized, also defines a global time in that same system. When Einstein, with the equivalence principle in mind, began investigating the behavior of clocks in accelerated frames, he found out that this simple connection between local and global time is lost.

In 1907, Einstein wrote a review paper titled “On the Relativity Principle and the Conclusions Drawn From It” [3]. In the final part of this review, he posed the question of whether the relativity principle could be generalized to include accelerated frames of reference. To explore this, Einstein compared two reference frames: an inertial system $\Sigma_{2}$ with a homogeneous gravitational field causing a free-fall acceleration γ along the negative *X*-axis, and another frame $\Sigma_{1}$ uniformly accelerating in empty space with an acceleration γ along the positive *X*-axis. Einstein noted that, for mechanical processes, the behaviors observed in $\Sigma_{1}$ do not differ from those in $\Sigma_{2}$: all bodies experience equal acceleration in a gravitational field, so the motions in $\Sigma_{2}$ are identical to those in $\Sigma_{1}$.

Based on this observation, Einstein proposed that *all* physical processes occur in exactly the same way in $\Sigma_{1}$ and $\Sigma_{2}$. Thus, an inertial system with a homogeneous gravitational field can be considered equivalent to an accelerated frame of reference without a gravitational field. This marked the first appearance of the celebrated principle of equivalence in the literature. It is important to stress that this principle is a hypothesis extending beyond the mechanical evidence of the equality of gravitational and inertial mass. It is applied to all physical phenomena, including electromagnetic phenomena like the propagation of light.

The equivalence principle allows predictions about what will happen in a gravitational field by considering the corresponding accelerated frame, where there is no gravitational field. The physics in an accelerated frame can be addressed using the special theory of relativity. In the 1907 paper, Einstein, therefore, examined the spatiotemporal relations between an inertial system *S* and a system Σ accelerating along the *X*-axis of *S* with constant acceleration γ. Both systems are equipped with measuring rods and clocks of identical construction. At *S*-time t=0, the frames *S* and Σ coincide, with vanishing instantaneous mutual velocity. At that moment, every clock in Σ is synchronized to show the same time as the clock in *S* with which it momentarily coincides. The time shown by the local clocks in Σ after this initial synchronization with *S* defines the “local time” σ of Σ.

As Einstein notes, in terms of this local time, local descriptions of physical processes will have the same form throughout system Σ. Indeed, acceleration does not systematically affect lengths and times, so that local descriptions in the accelerated frame match those from local inertial systems that are instantaneously comoving with each clock. Of course, in general, acceleration does affect rods and clocks: e.g., a wristwatch that falls to the ground could well cease to function. However, the importance of such acceleration effects will depend on the nature of the clock. The idea behind the statement that there is no *systematic* metric effect of acceleration is that it is possible to find physical systems that are so robust or insensitive that acceleration effects can be neglected. This assumption is known as the Clock Hypothesis, and defines ideal clocks.

However, Einstein warned that we should not regard σ as “the time of system Σ”, because the accelerating local clocks of Σ will fall out of sync with each other (according to the 1905 criterion). As seen from *S*, all clocks in Σ undergo exactly the same accelerated motion, meaning that, at any instant of *S*-time, all Σ-clocks will have ticked the same amount of time. Therefore, they will remain synchronized from *S*’s perspective. However, this synchronization will not hold when viewed from an inertial system $S^{\prime }$ that is instantaneously at rest relative to Σ at any *S*-time t>0: at such an instant, Σ, and therefore, also, $S^{\prime }$, will have a non-zero velocity relative to *S*. Consequently, the simultaneity relations in *S* and $S^{\prime }$ must differ, as dictated by special relativity.

In addition to the local times σ, Einstein, therefore, introduces a “global time” in Σ, which is essentially the time of the instantaneously comoving inertial system $S^{\prime }$. Specifically, the global time τ of an event in Σ is the time indicated by the local clock at the origin of Σ at the moment that is simultaneous with the event, according to the simultaneity of the instantaneously comoving inertial system $S^{\prime }$. Because local clocks are synchronous according to the simultaneity of *S*, and global clocks according to the different simultaneity of $S^{\prime }$, the local time σ of Σ and the global time τ of Σ are not the same.

The quantitative relation between σ and τ can be determined with the help of the special relativistic transformation formulas. Two events that take place at positions $x_{1}$ and $x_{2}$ and times $t_{1}$ and $t_{2}$ of *S*, respectively, are simultaneous with respect to $S^{\prime }$ (and, therefore, with respect to Σ) if $t_{1}-x_{1}v/c^{2}=t_{2}-x_{2}v/c^{2}$, where *v* is the speed of $S^{\prime }$ with respect to *S*. If the time difference with t=0 is small, and if the velocity *v* is small as well, we have in first approximation $x_{2}-x_{1}=x_{2}^{\prime }-x_{1}^{\prime }=\xi_{2}-\xi_{1}$, with ξ the coordinate in system Σ along the common *X*-axis. Moreover, we have in this approximation $t_{1}=\sigma_{1}$, $t_{2}=\sigma_{2}$, and v=γτ.

It follows that $\sigma_{2}-\sigma_{1}\approx \left(\xi_{2}-\xi_{1}\right)\left(\gamma \tau \right)/c^{2}$, if $\sigma_{2}$ and $\sigma_{1}$ correspond to the same value of τ. When we take the origin of the coordinate system as the place of the first event, so that $\sigma_{1}=\tau$ and $\xi_{1}=0$, we obtain:

(1) $$\sigma =\tau (1+\frac{\gamma \xi }{c^{2}}).$$

According to the principle of equivalence, the same equation holds in a system in which there is a homogeneous gravitational field. In that case, we can replace γξ with the gravitational potential Φ, so that we obtain

(2) $$\sigma =\tau (1+\frac{\Phi }{c^{2}}).$$

Summing up, there are two timekeeping systems in Σ: a local one and a global one. Both the local time σ and the global time τ can be indicated by a set of clocks. The local time is shown by clocks that are initially synchronized with the clocks in *S* and then move along with Σ without further adjustments. The global time of Σ is shown by clocks synchronized according to the Einstein synchrony of the instantaneously comoving frame $S^{\prime }$, with the clock at the origin of the comoving frame set to show the same time as the σ-clock at the origin of Σ.

When we are interested in local processes and measurements, it is natural to use the σ-clocks, which run freely without synchronization interventions. However, when describing extended processes, such as the propagation of signals, we need a notion of simultaneity to coordinate events at different positions. In this case, the τ-clocks are useful.

From Equation (2), it follows that, at global instant τ, a local clock at a position with gravitational potential Φ indicates a time that differs from the time of a local clock at the origin (where Φ=0) by a factor of $\left(1+\Phi /c^{2}\right)$. This difference is experimentally significant: a stationary observer at some other position in Σ will directly *see* that the two local clocks tick at different frequencies, differing by exactly this factor.

This is an essential point, which will play a key role in our later explication of Einstein’s derivation of the general relativistic redshift formula. Its background is that the time Δτ needed for light to travel from a fixed source in Σ to a stationary observer is independent of τ. As Einstein explains ([3], p. 458), the definition of τ does not single out any special point in time, although it does involve the arbitrary choice of a spatial origin. Therefore, the laws of nature cannot depend explicitly on τ, though they may be position-dependent. It follows that a light signal that has a certain duration during its emission, expressed in τ, will have the same duration, again expressed in τ, when received by an observer. Put differently, intervals of global time τ are invariant under transport by light signals.

On the other hand, the laws governing local processes take their standard local special relativistic form when expressed by means of the local time σ, and this standard form is independent of spatio-temporal location. So, the σ-locally measured frequency of light emitted by a localized standard source (e.g., a spectral line) will be position-independent. However, the relation between τ and σ varies with position, so that signals from sources at different positions, with the same duration in terms of τ and consisting of light emitted with the same natural frequency as measured by local σ clocks, may have different numbers of wavelengths contained in them. An observer will be able to compare such signals of equal τ-duration and establish their frequency difference.

This remarkable result leads to the prediction that clocks of identical construction, but at positions with different gravitational potentials will be observed to run at different rates. As Einstein expressed it, clocks at higher values of the gravitational potential run faster than clocks lower in the gravitational field. This statement may seem paradoxical, since local measurements at the positions of the clocks will surely fail to reveal any differences in clock rates. But, the statement is elliptical: as just explained, it is about the comparison of two clocks from a different third position, and this dissolves the paradox (cf. the discussion in Section 5).

An example of the influence of gravity on the rate of clocks is provided by the behavior of atoms and molecules emitting spectral lines. From the aforementioned argument, it can be concluded that spectral light coming from the surface of the Sun will arrive on Earth with a frequency slightly shifted to the red end of the spectrum (when compared to the frequency of the same spectral line emitted on Earth).

Another prediction is that light will be bent in a gravitational field ([3], p. 461). The derivation of this effect in the 1907 paper is rather complex. Additionally, the influence of gravity on clocks can be demonstrated in a simpler way than explained above. For these reasons, along with the opportunity for new experimental tests, Einstein returned to this topic in 1911.

## 3. Gravity, Time, and Light in 1911

The principle of equivalence again takes a central place in the 1911 paper [4]. As Einstein shows, a simple thought experiment using the equivalence principle suffices to derive how clocks behave in a gravitational field.

Let there be two bodies *A* and *B* in a system of reference *K* in which there is a homogeneous gravitational field. Both bodies, assumed to be very small, are located on the *z*-axis of *K*; the acceleration due to gravity is −γ (directed downward in the *z*-direction). *B* is located higher in the field, at a distance *h* from *A*, so that the gravitational potential at *B* is greater than at *A*, Φ(B)−Φ(A)=γh. *B* emits electromagnetic radiation in the direction of *A*, and we are interested in how gravity influences this signal traveling from *B* to *A* (see Figure 5 in [13], p. 102).

The principle of equivalence tells us that we can replace system *K* with a system $K^{\prime }$ that possesses a constant acceleration γ in the positive *z*-direction and in which there is no gravitational field. In order to have a situation that is equivalent to the original one, we have to assume that *A* and *B* are located at fixed positions on the $z^{\prime }$-axis, with constant mutual distance *h*. Finally, let $K_{0}$ be an inertial system without gravitation that, at the moment of the emission of the radiation, is instantaneously at rest with respect to $K^{\prime }$.

When we describe the process of the emission, propagation, and reception of the radiation from system $K_{0}$, *B* has no velocity relative to $K_{0}$ when the radiation is emitted and the radiation takes a time h/c to arrive at *A* (in first approximation). *A* possesses the approximate speed (hγ)/c=v when the radiation arrives at its position. Now, Einstein notes, if the radiation had the frequency $\nu_{2}$ when it was emitted by *B*, as measured by a standard clock positioned near *B* and comoving with *B*, the radiation received in *A* will have a different frequency $\nu_{1}$ as measured by a standard clock comoving with *A*. Indeed, when the signal arrives at *A*, *A* (and its clock) will possess a speed *v* relative to $K_{0}$, so that there will be a change in measured frequency on account of the Doppler effect. The relation between $\nu_{1}$ and $\nu_{2}$ is given by the Doppler formula:

(3) $$\nu_{1}=\nu_{2}\left(1+\frac{\gamma h}{c^{2}}\right).$$

According to the equivalence principle, this same result should be valid in system *K*, where a homogeneous gravitational field is present. This means that light emitted at a higher value of the gravitational potential will arrive at positions with a lower potential with a higher frequency (as measured by a local clock). Rewriting Equation (3), we find

(4) $$\nu_{1}=\nu_{2}\left(1+\frac{\Phi }{c^{2}}\right),$$

where Φ is the gravitational potential at *B* and the value of the potential at *A* has been set to 0. This is the same gravitational redshift formula as derived in the 1907 paper, Equation (2).

The 1911 derivation of the redshift formula may seem of a completely different nature from that in the 1907 paper: in 1907, Einstein emphasized the necessity of a global time, whereas the 1911 derivation appears to involve only local times, measured by clocks of the same kind located at different positions in the gravitational field. However, that impression is deceptive. Equation (4) implies that the frequency of the traveling light increases between *B* and *A*. But, this, Einstein notes, seems paradoxical: how could the number of beats per second change during the light’s journey, given that the source continually emits light of constant frequency, in a stationary process? Einstein discusses the problem as follows ([4], pp. 905–906; pp. 105–106 in the English translation):

On superficial consideration Equation (4) seems to assert an absurdity. If there is constant transmission of light from *B* to *A*, how can any other number of periods per second arrive at *A* than is emitted from *B*? But the answer is simple. We cannot regard $\nu_{2}$ or respectively $\nu_{1}$ simply as frequencies (as the number of periods per second) since we have not yet determined a time in system *K*. What $\nu_{2}$ denotes is the number of periods per second with reference to the time-unit of the clock *U* at *B*, while $\nu_{1}$ denotes the number of periods per second with reference to the identical clock at *A*. Nothing compels us to assume that the clocks *U* in different gravitation potentials must be regarded as going at the same rate. On the contrary, we must certainly define the time in *K* in such a way that the number of wave crests and troughs between *B* and *A* is independent of the absolute value of time: for the process under observation is by nature a stationary one. ... Therefore the two clocks at *A* and *B* do not both give the “time” correctly. If we measure time at *A* with the clock *U*, then we must measure time at *B* with a clock which goes $1+\Phi /c^{2}$ times more slowly than the clock *U* when compared with *U* at one at the same place. For when measured by such a clock, the frequency of the light-ray which is considered above is at its emission from *B* given by $\nu_{2}\left(1+\Phi /c^{2}\right)$, and is therefore, by (4), equal to the frequency $\nu_{1}$ of the same light-ray on its arrival at *A*.

Einstein here explicitly introduces a global time in system *K* that differs from what local clocks indicate. This global time corresponds to the time τ defined in the 1907 article, while the clocks at *A* and *B* correspond to the local times σ. As in the 1905 and 1907 articles, both the local and global times are assumed to be directly measured by sets of clocks. In the 1907 article, this material implementation of global time was realized by using the Einstein synchrony of instantaneously comoving inertial systems, whereas in the 1911 paper, the clocks indicating global time are introduced directly, via the rule that they be constructed such that they tick $1+\Phi /c^{2}$ times more slowly than local clocks at the same location.

In the *Collected Papers of Einstein, Volume 3*, the editors comment that Einstein’s train of thought in the 1911 paper is quite different from the one in 1907 ([14], p. 497). In particular, they claim that the “corrected” clocks just mentioned in the quoted passage of the 1911 paper do not figure in the 1907 article, and they refer to Pais’ biography of Einstein ([15], pp. 198–199) for a more extensive analysis. However, we have just seen that the corrected local clocks show the global time in *K*, and as discussed in Section 2, this same concept of global time played a major role in the 1907 paper. Moreover, the indicated passage in Pais’ biography states that local clocks cannot be assumed to run at equal rates, but does not mention Einstein’s correction procedure for calculating the “real”, i.e., global time. It, thus, remains unclear how the assertion that the local clocks do not run at the same rate should be interpreted.

The global time in *K*, introduced by Einstein in the above quotation, is such that the frequency of traveling light, during its trip, remains constant when expressed in it. In other words, global time intervals are invariant under transport by light signals. However, the relation between global time and the time shown by local clocks is position-dependent, so that the frequency of the traveling light, as determined by local clocks, varies with location. Seen from this perspective, the 1911 redshift explanation follows exactly the same pattern as the 1907 explanation.

The 1911 paper ends with a calculation of the bending of light in a gravitational field and is most famous for this prediction. This calculation is based on the observation that the velocity of light, measured in global time, will not be constant, but will vary with the gravitational potential according to

(5) $$c=c_{0}\left(1+\frac{\Phi }{c^{2}}\right),$$

where $c_{0}$ is the value at the origin (where Φ=0). Huygens’s principle, applied to this situation with a variable speed of light, implies that light rays will be deflected in the direction in which the gravitational potential decreases. For a ray grazing the Sun, Einstein finds a deflection of $0.83^{\prime \prime }$, and comments ([13], p. 108) that, “it would be a most desirable thing if astronomers would take up the question here raised”. (The value found by Einstein in 1911 reflects the influence of gravity on time, but does not take into account that gravity also deforms the spatial geometry. The full general theory of relativity predicts a value that is twice the value predicted by the 1911 considerations.)

## 4. The Final Theory of General Relativity, 1916

In the first part of his 1916 overview of the just-finished general theory of relativity [9], Einstein paid a great deal of attention to the conceptual foundations of his new theory. To make clear that non-Euclidean spatial relations must be expected in the presence of gravitational fields, and that time will have unusual properties as well, Einstein discusses the example of a frame $K^{\prime }$ that rotates (and, therefore, accelerates) with respect to an inertial frame *K* ([13], pp. 115–116). Concerning time in the rotating frame, he writes, after having discussed the impossibility of retaining Euclidean geometry (translation following [13], with minor adjustments):

Neither can we introduce a time in $K^{\prime }$ that meets the physical requirements if this time is to be indicated by clocks of identical construction at rest relatively to $K^{\prime }$. To see this, let us imagine two such identical clocks, placed one at the origin of the coordinates and the other at the circumference of the circle and both considered from the “stationary” frame *K*. By a familiar result of the special theory of relativity, the clock at the circumference—judged from *K*—goes more slowly than the other, because the former is in motion and the other at rest. An observer at the common origin of coordinates, capable of seeing the clock at the circumference by means of light, would therefore see it lagging behind the clock beside him. As he will not make up his mind to let the velocity of light along the path in question depend explicitly on the time, he will interpret his observations as showing that the clock at the circumference “really” goes more slowly than the clock at the origin. So he will be obliged to define time in such a way that the rate of a clock depends upon where the clock may be.

There is a striking similarity here to Einstein’s reasoning in his 1907 and 1911 articles. The idea is that local clocks moving along with an accelerated system, and thus clocks stationary in a system with a gravitational field, will indicate bona fide local times. However, local times at different positions will not combine into one physically acceptable global time (cf. Section 2 in [16], Sections 5.2–5.3 in [17]). According to Einstein, an acceptable global time should ensure that physical laws, particularly the law governing the propagation of light, do not depend explicitly on time.

The importance of the time independence of the speed of light, measured in global time, lies in its enabling a direct and simple comparison of clocks at different positions. Indeed, if the speed of light does not depend on time, the time interval required for light to travel between two fixed points remains constant. Therefore, if both a source and receiver are stationary with respect to a reference system, the global time interval between two emission events will equal the global time interval between the corresponding reception events. Light signals, thus, transport global time intervals without change. An observer can directly measure the global time intervals between distant emission events and check whether distant processes occur more slowly or quickly (with respect to global time) compared to similar processes at their own location. In this way, the observer can verify if distant clocks “really” tick slower or faster than their own clock.

In the concrete examples that we have seen, both local and global time were associated with sets of clocks. Local time is measured by stationary clocks that keep ticking without external intervention, while global time is measured by stationary clocks that undergo suitable adjustments (that may be quite complicated). In the case of the rotating disk, the global clocks may be chosen so that they indicate the time of the inertial clocks with which they momentarily coincide. However, the only really important point is that global time is defined such that the laws of nature do not explicitly depend on time. In this case, global time intervals are transported unchanged by physical signals, and this property makes the comparison of processes taking place at different positions straightforward. The invariance of transported global time intervals is the leitmotif running through all the articles we have looked at.

In the formal part of the 1916 paper, things become abstract and general. Specifically, the restriction to homogeneous gravitational fields is explicitly dropped. (In his earlier work Einstein had mentioned only en passant his assumption that his results for homogeneous fields remained valid in the general case). In general, there now no longer exist physically privileged global frames that in an obvious way can be associated with global clocks. Nevertheless, Einstein continued to use his earlier way of reasoning in paradigm cases, in order to illustrate the application of his final theory. The discussion of the gravitational redshift at the end of the 1916 review paper is a case in point.

The last section of the 1916 review, entitled “Behaviour of Rods and Clocks in the Static Gravitational Field. Bending of Light-rays. Motion of the Perihelion of a Planetary orbit”, is devoted to crucial and testable predictions of the new theory ([13], pp. 160–164). The influence of gravity on clocks and the gravitational redshift are dealt with very quickly. For a unit clock that is at rest in a static gravitational field, we have for one clock period ds=1 and $dx_{1}=dx_{2}=dx_{3}=0$. Therefore, $g_{44}{dx_{4}}^{2}=1$, so that $dx_{4}=1/\sqrt{g_{44}}$. If there is a point mass with mass *M* at the origin of the coordinates, the general relativistic field equations tell us that, in first approximation, $g_{44}=1-\kappa M/4\pi r$, with κ the gravitational coupling constant appearing in these equations ($\kappa =8\pi G/c^{2}$, with *G* Newton’s constant) and *r* the radial spatial distance from the point mass. Therefore,

(6) $$dx_{4}\approx 1+\frac{\kappa M}{8\pi r}.$$

Einstein immediately concludes ([13], p. 162):

Thus the clock goes more slowly if set up in the neighbourhood of ponderable masses. From this it follows that the spectral lines of light reaching us from the surface of large stars must appear displaced towards the red end of the spectrum.

Einstein’s 1916 reasoning concerning the rate of clocks and the gravitational redshift closely follows his earlier discussions, as we will discuss in a moment. As we will see, not taking this historical background into account can easily lead to misunderstandings.

## 5. Criticisms of the Early Redshift Derivations

Einstein’s 1916 derivation of the gravitational redshift soon became standard; it can still be found in present-day textbooks. An important role in making it widely known and popular was played by the work of Eddington. Eddington was the first to make the general theory of relativity known in the English-speaking world, and his seminal publications, Report on the Relativity Theory of Gravitation (1918) [18] and Space, Time and Gravitation (1923) [19], were widely read. Of these two publications, especially the less technical Space, Time and Gravitation was very influential. In this book, Eddington discusses the comparison of frequencies emitted by atoms of the same kind, but located at different positions, e.g., on the Sun and on Earth, respectively. Eddington explains the situation as follows ([19], pp. 128–129, italics in the original; Eddington used γ instead of $g_{44}$, and employed units in which κ/8π=1):

Consider an atom momentarily at rest at some point in the solar system... If ds corresponds to one vibration ... we have $ds^{2}=g_{44}dt^{2}$. The *time* of vibration dt is thus $1/\sqrt{g_{44}}$ times the *interval* of vibration ds.

Accordingly, if we have two similar atoms at rest at different points in the system, the interval of vibration will be the same for both; but the time of vibration will be proportional to the inverse square-root of $g_{44}$, which differs for the two atoms. Since $g_{44}=1-\frac{2M}{r}$, $\frac{1}{\sqrt{g_{44}}}=1+\frac{M}{r}$, very approximately.

Take an atom at the surface of the Sun, and a similar atom in a terrestrial laboratory. For the first, 1+M/r = 1.00000212, and for the second 1+M/r is practically 1. The time of vibration of the solar atom is thus longer in the ratio 1.00000212, and it might be possible to test this by spectroscopic examination.

There is one important point to consider. The spectroscopic examination must take place in the terrestrial laboratory; and we have to test the period of the solar atom by the period of the waves emanating from it when they reach the Earth. Will they carry the period to us unchanged? Clearly they must. The first and second pulse have to travel the same distance *r*, and they travel with the same velocity dr/dt; for the velocity of light in the mesh-system used is 1−2M/r, and though this velocity depends on *r*, it does not depend on *t*. Hence the difference dt at one end of the waves is the same as that at the other end.

This account closely follows Einstein’s 1916 derivation, with the difference that Eddington has added an explanation of why the (global) time interval dt is invariant under transport by light signals. This explanation echoes Einstein’s reasoning in his 1907 and 1911 papers, and his discussion of the rotating frame in the 1916 paper. It is what we have called the leitmotif of Einstein’s arguments involving global time. In his earlier Report ([18], pp. 56–58), Eddington had presented exactly the same derivation of the redshift formula, but without the passage about the invariance of dt. Since the publication of the Report, erroneous statements about the role of dt and ds in the explanation of the gravitational redshift had appeared in the literature, to which Eddington had responded immediately by emphasizing that the duration dt of a process has the same value, both at the source and at the position of the receiver ([5], p. 174). Apparently, this episode prompted him to add the explanatory lines reported above to his earlier derivation, when he wrote [19]. The alternative suggestion that, in 1918, Eddington mistook dt for the measured proper time ds [5,7,11,12] seems implausible: following his general relativistic derivation, Eddington reproduced both Einstein’s Doppler and rotating disk arguments ([18], pp. 56–58), so that it is almost certain that he knew of Einstein’s remarks about the use of global time—but more about this issue in a moment.

In an influential article published in 1980, John Earman and Clark Glymour [11] criticized the early derivations of the gravitational redshift by Einstein, Eddington, and others. They characterize Einstein’s 1907 derivation as cumbersome and obscure, lacking clarity regarding the meanings of “time” and “local time” ([11], p. 178), without going into details. They also discuss the 1911 paper and describe the thought experiment in which radiation is emitted from *B* to *A* in a homogeneous gravitational field. As detailed in Section 3 and mentioned by Earman and Glymour, Einstein concluded that clocks at different positions run at different rates (measured in global time), implying that the velocity of light is position-dependent (as measured in global time). Earman and Glymour comment ([11], pp. 181–182),

All of the heuristic derivations of the red shift can be faulted on various technical grounds. But to raise such objections is to miss the purpose of heuristic arguments, which is not to provide logically seamless proofs but rather to give a feel for the underlying physical mechanisms. It is precisely here that most of the heuristic red shift derivations fail—they are not good heuristics. For they are set in Newtonian or special relativistic space-time; but the red shift strongly suggests that gravitation cannot be adequately treated in a flat space-time. Einstein’s resort to the notions of a variable speed of light and variable clock rates in a gravitational field can be seen as an acknowledgment, albeit unconscious, of this point; but as we will now see, these notions served to obscure the role of curvature of space-time as the light ray moves from source to receiver.

It is true that the 1907 and 1911 papers do not go beyond using the principle of equivalence in Newtonian or Minkowski spacetime and are, therefore, flawed from the point of view of the finished general theory of relativity. There is no principled discussion of inhomogeneous gravitational fields in the 1907 and 1911 papers, although Einstein does conjecture in several places that his results for homogeneous fields will also apply to inhomogeneous ones. But, it appears odd to dismiss Einstein’s arguments as bad heuristics on these grounds. Good heuristics usually retain major parts of old conceptual frameworks and add some new idea or perspective to suggest results that should be rigorously derived within a new theory. Einstein’s heuristics did exactly that.

More importantly, Earman and Glymour level a specific technical objection to Einstein’s and Eddington’s derivations, which they see as devastating. They claim that the derivations in question do not pay any attention to the process by which information is transmitted from the source to the receiver and, therefore, can only be misleading ([11], p. 176). After quoting Einstein’s 1916 derivation of Equation (6) and his conclusion (“The clock goes more slowly if set up in the neighbourhood of ponderable masses. From this it follows that the spectral lines of light reaching us from the surface of large stars must appear displaced towards the red end of the spectrum.”), Earman and Glymour comment ([11], pp. 182–183),

To the modern eye, Einstein’s derivation is no derivation at all, for the Formula (6) expresses only a co-ordinate effect [...] Einstein provided no deduction from the theory to explain what happens to a light ray or photon as it passes through the gravitational field on its way from the Sun to the Earth. Unfortunately, Einstein’s ‘derivation’ was dressed up by the expositors of the general theory, and it quickly became codified in the literature as the official derivation.

Earman and Glymour go on to highlight Eddington’s role in spreading Einstein’s approach, and object to Eddington’s redshift derivation in the Report on the same grounds for which they objected to Einstein’s derivation. They claim that both Einstein and Eddington confused coordinate time with proper time and failed to engage with the crucial question of how the radiation emitted at the Sun is received on Earth.

We have already commented, a moment ago, on the implausibility of Earman’s and Glymour’s reading of Eddington’s text according to which he simply conflated dt and ds. Moreover, any thought that Einstein in his early derivations forgot about the process of transmission between source and observer is patently incorrect. It was exactly the properties of this transmission process, in particular that it leaves global time intervals unchanged, that formed the leitmotif of Einstein’s reasoning in his early work. As we have seen, the essential point in these arguments was that the speed of light does not depend on global time, so that consecutive light signals need the same global time interval, over and over again, to travel from sender to receiver. This condition on global time, that it make descriptions of stationary processes time-independent, is clearly fulfilled in the case of static gravitational fields (the context of Einstein’s 1916 derivation); it is also precisely what is emphasized by Eddington in his Space, Time and Gravitation, in the passage reproduced above.

Earman and Glymour present their own (correct) derivation of the redshift formula by arguing at some length that, in a static gravitational field, coordinates can be chosen in such a way that the coordinate time interval is transmitted without change. Via a rather roundabout use of this premise, they finally arrive at a formula that is equivalent to Einstein’s ([11], pp. 184–185). The argument is basically equivalent to Eddington’s in Space, Time and Gravitation.

The criticism by Earman and Glymour has set the tone for later critical analyses of Einstein’s early work on general relativity. Generally, Einstein’s 1916 derivation (and his subsequent unmodified presentations of the same derivation) is condemned as being marred by confusion. Eddington’s 1918 derivation shares the same fate.

For instance, Giovanelli ([7], pp. 41–42) calls Einstein’s 1916 derivation, “puzzling”, and follows Earman and Glymour in criticizing Einstein’s use of *t* as if this coordinate had a physical meaning. Bacelar Valente [12], declaring that he takes Earman and Glymour’s article as his, “benchmark”, claims that both Einstein and Eddington conflated ds and dt. Darrigol ([5], pp. 172–173) characterizes Einstein’s 1916 redshift derivation as “very misleading, problematic, and confusing,”; although he sees extenuating circumstances in a possible connection to Einstein’s earlier work (thus moving in the direction of our own analysis). Darrigol ([5], p. 174) also criticizes Eddington’s 1918 derivation for not discussing the signals that travel from one clock to another.

Moreover, all three of these recent authors take offense at Einstein’s claim that clocks run slower near ponderable masses. They argue that this is incorrect, because local measurements will show no deviations whatsoever from the clock’s normal rate. However, as we have seen, Einstein took great care in explaining that the slowing down of clocks is a *comparative* effect, pertaining to the comparison of clocks at different positions (with global time acting as an intermediary). This criticism is, therefore, similar to questioning the Lorentz contraction of moving rods on the grounds that a comoving rod will show no discrepancy at all.

The background of these misunderstandings seems to be that, if one looks at Equation (6) with modern eyes, the only thing one sees is a statement about an arbitrary and uninterpreted coordinate. In modern expositions of general relativity, coordinates are considered to be conventional markers of events; in particular, the time coordinate $x_{4}$ is not required to have a physical interpretation in terms of clocks or any other physical process. (There are exceptions. For example, in [17], p. 22, coordinates are associated with *coordinate clocks*, and the local clocks in a static gravitational field are said to run slow, because they lag behind coordinate clocks at the same locations [17], p. 219). Only after a solution of the field equations has been obtained, and an expression for $ds^{2}$ in terms of coordinates has been found, does it make sense to ask about the meaning of the coordinates in terms of measured length and time intervals. But Einstein did not approach the subject from this modern perspective. Although, in his definitive work on general relativity, Einstein took an important step towards the modern view by acknowledging that arbitrary coordinate systems may be used ([9], p. 776), the fruitful ideas of his earlier heuristic work continued to play a major role in his thinking. In our particular case, he adhered—as far as possible—to the notion of global time in which physical laws are time-independent. Seen that way, the physical properties of *t* during signaling processes in static gravitational fields are obvious.

## 6. Conclusions: Coordinates and Time

In 1921, five years after his review article on the general theory of relativity, Einstein delivered the Stafford Little Lectures at Princeton University. These lectures, published in 1923 as The Meaning of Relativity [10], covered both the special and general theories of relativity. Revisiting the behavior of rods and clocks in a gravitational field ([10], pp. 90–92), Einstein observed that only in local inertial systems can coordinates be chosen to match “naturally measured lengths and times.” In the symmetric case of a static field generated by a central mass, with coordinates adapted to the global symmetry, a unit measuring rod will not always match a unit coordinate interval. As Einstein explained, its “coordinate length” will be shortened. He further noted that this coordinate length, and its variation based on location and orientation, depends on the chosen coordinate system. So, in modern terms, this is purely a coordinate effect.

Regarding time, Einstein remarked that the interval between two beats of a unit clock (ds=1) corresponds to a longer “time” ($dx_{4}>1$), “in the unit used in our system of coordinates”. He then continued ([10], p. 92),

The rate of a clock is accordingly slower the greater is the mass of the ponderable mass in its neighbourhood. We therefore conclude that spectral lines which are produced on the Sun’s surface will be displaced towards the red, compared to the corresponding lines produced on the Earth, by about $2.10^{-6}$ of their wave-lengths.

This argument is almost word for word the same as the one occurring in the 1916 paper. As we have seen, modern critics have accused Einstein of conflating, in this argument, ds and dt and of forgetting about the process of information transfer between clocks. But, remarks made by Einstein in correspondence, to be discussed in a moment, document that there can be no doubt that, in 1921, he was fully aware of the meaning of *t*, in particular concerning its role in describing the passing of signals from source to observer. These remarks by Einstein confirm our analysis.

In the summer of 1920, a year before the Princeton lectures, Einstein got involved in a correspondence with Edouard Guillaume, an old colleague from Einstein’s days at the Bern patent office. Guillaume had developed into a fierce opponent of relativity theory, and a defender of absolute time. Although Einstein made no secret of his opinion that Guillaume’s writings were incomprehensible, nonsensical, and even, “crazy”, he felt obliged to respond collegially and seriously, and to explain basic points of relativity as clearly and simply as possible. In a letter of 19 July 1920, he wrote to Guillaume ([20], p. 339; my translation),

initially, dt has only a purely conventional meaning in the general theory of relativity. But when we consider the problem of the shift of spectral lines, *t* once again takes on a kind of absolute meaning. This is due to the fact that the four coordinates are chosen in such a way that the field of an isolated point mass assumes a static form; in other words, in such a way that the number of wavelengths traveling between the Sun and the Earth cannot depend on *t*.

This constancy of the frequency when expressed in *t* is exactly the core of what we have called the leitmotif of Einstein’s redshift reasoning. The quotation makes it virtually certain that Einstein, when presenting his 1916 derivation, had the invariance of dt under transport by light signals in the back of his mind, as an obvious point.

When working on general relativity, Einstein had certainly become well aware that coordinates are, in principle, arbitrary labels [7]. This is also clear from his above-mentioned discussion of measuring rods. Nevertheless, Einstein maintained his earlier strategy of making the concept of time as physically tangible as possible, given the concrete problem he considered. This approach had significantly aided him in developing the special theory of relativity and in taking the initial steps towards general relativity. In specific cases with global symmetries, such as static gravitational fields, the strategy of introducing a physically meaningful global time remained helpful even in the final form of general relativity.

It remains true that a number of important general ideas that motivated Einstein’s heuristic work could not be retained in the final theory of general relativity; see, e.g., [21,22]. Nevertheless, there is a strong continuity in Einstein’s work from 1907 to 1916 and beyond. Recognizing this continuity restores coherence and transparency to a number of arguments that otherwise may seem muddled or mysterious, and that have led to confusion in the literature.

## Data Availability

No new data were created or analyzed in this study. Data sharing is not applicable to this article.
